# Supplementary material for: Marine protected areas promote stability of reef fish communities under climate warming
Source: Nat Commun. 2024 Feb 28;15:1822. doi: 10.1038/s41467-024-44976-y (PMC10902350; doi:10.1038/s41467-024-44976-y)
Supplement: Supplementary file 1 — Supplementary Information [file 41467_2024_44976_MOESM1_ESM.pdf]

Supplementary Information for:

MARINE PROTECTED AREAS PROMOTE STABILITY OF REEF FISH COMMUNITIES UNDER CLIMATE WARMING

Lisandro Benedetti-Cecchi[1*], Amanda E. Bates[2], Giovanni Strona[3], Fabio Bulleri[1], Barbara Horta e Costa[4], Graham J. Edgar[5,6], Bernat Hereu[7], Dan C. Reed[8], Rick D. Stuart-Smith[5,6], Neville S. Barrett[5], Kushner J. David[9], Michael J. Emslie[10], Jose Antonio Garcia-Charton[11], Emanuel J. Gonçalves[12,13], Eneko Aspillaga[14].

[1]Department of Biology, University of Pisa, URL CoNISMa, Via Derna 1, Pisa, Italy. [2]Department of Biology, University of Victoria, Canada. [3]European Commission, Joint Research Centre, Ispra, Italy. [4]CCMAR, Centre of Marine Sciences, University of Algarve, Building 7, Faro, 8005-139, Portugal. [5]Institute for Marine and Antarctic Studies, University of Tasmania, Hobart, Tasmania, Australia. [6]Reef Life Survey Foundation, Battery Point, Tasmania, Australia. [7]Departament de Biologia Evolutiva, Ecologia i Ciències Ambientals, Facultat de Biologia, Institut de Recerca de la Biodiversitat (IRBIO), Universitat de Barcelona, Barcelona, Spain. [8]Marine Science Institute, University of California Santa Barbara, Santa Barbara, 93106, CA, United States. [9]Channel Islands National Park, Ventura, CA, United States. [9]Departamento de Ecología e Hidrología, Universidad de Murcia, Campus Espinardo, Murcia, 30100, Spain. [10]Australian Institute of Marine Science, Townsville, Queensland, Australia. [11]Departamento de Ecología e Hidrología, Universidad de Murcia, Campus Espinardo, Murcia, 30100, Spain. [12]MARE – Marine and Environmental Sciences Centre, ISPA – Instituto Universitário, Lisbon, Portugal. [13]Oceano Azul Foundation, Lisbon, Portugal. [14]Instituto Mediterráneo de Estudios Avanzados (IMEDEA, CSIC-UIB), 07190 Esporles, Spain.

*Corresponding author:
Lisandro Benedetti-Cecchi
lbenedetti@biologia.unipi.it

The file includes:

Supplementary Figures 1-15

Supplementary Tables 1-8

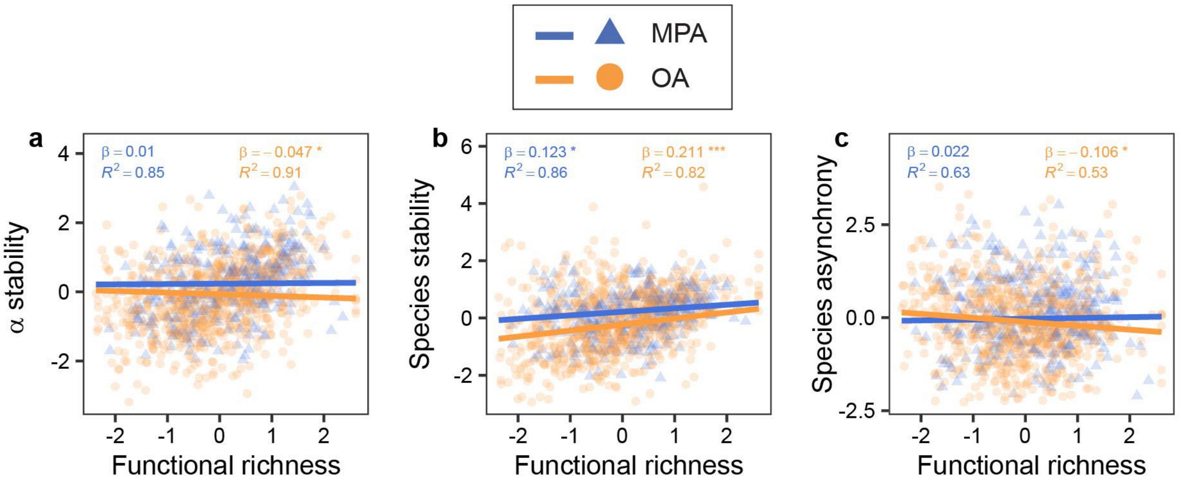

Supplementary Fig. 1. Relationships of alpha stability (**a**), species stability (**b**) and asynchrony (**c**) with functional richness. Data are shown as z-scores for marine protected areas (MPA, blue lines and symbols) and open areas (OA, orange lines and symbols). Panels include the regression parameters estimated from the Linear Mixed Effect Models, their significance (***, $p<0.001$; *, $p<0.05$) and the conditional coefficients of determination, which accounts for both fixed and random effects (R^2). Full statistical results are reported in Supplementary Table 1.

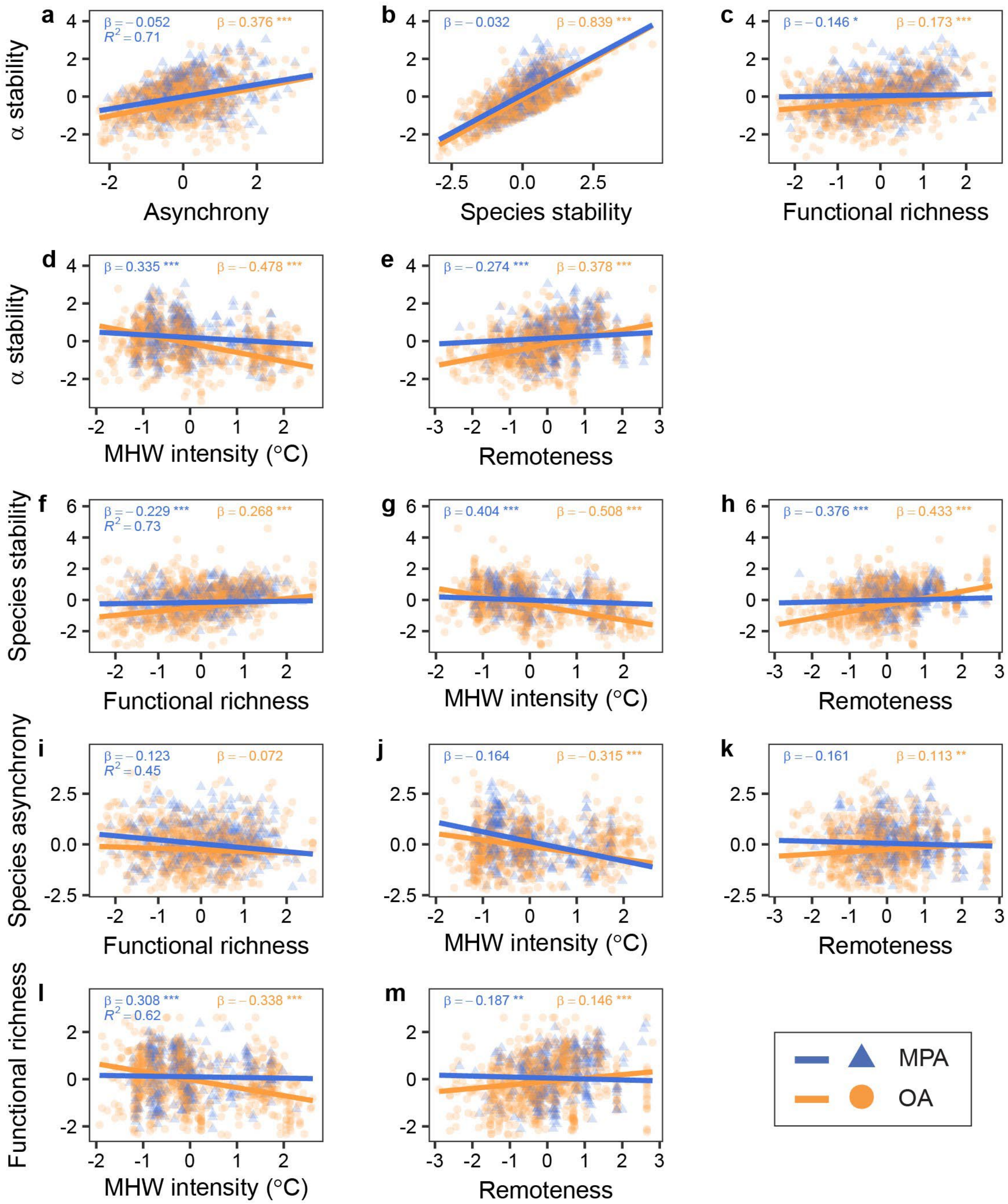

Supplementary Fig. 2. Full analysis of relationships between stability, asynchrony and their hypothesized drivers based on Linear Mixed Effect Models that included an interaction term between each predictor and the two levels of protection: marine protected areas (MPA, blue lines and symbols) and open areas (OA, orange lines and symbols). Data are shown as z-scores. Panels include the regression parameters estimated from mixed-effect models, their significance (***, p<0.001; **, p<0.01; *, p<0.05) and the conditional coefficients of determination, which accounts for both fixed and random effects (R^2, indicated only in the first panel for each response variable). Regression parameters for MPAs are the interaction terms indicating deviations from OA. Full statistical results are reported in Supplementary Tables 2 and 3.

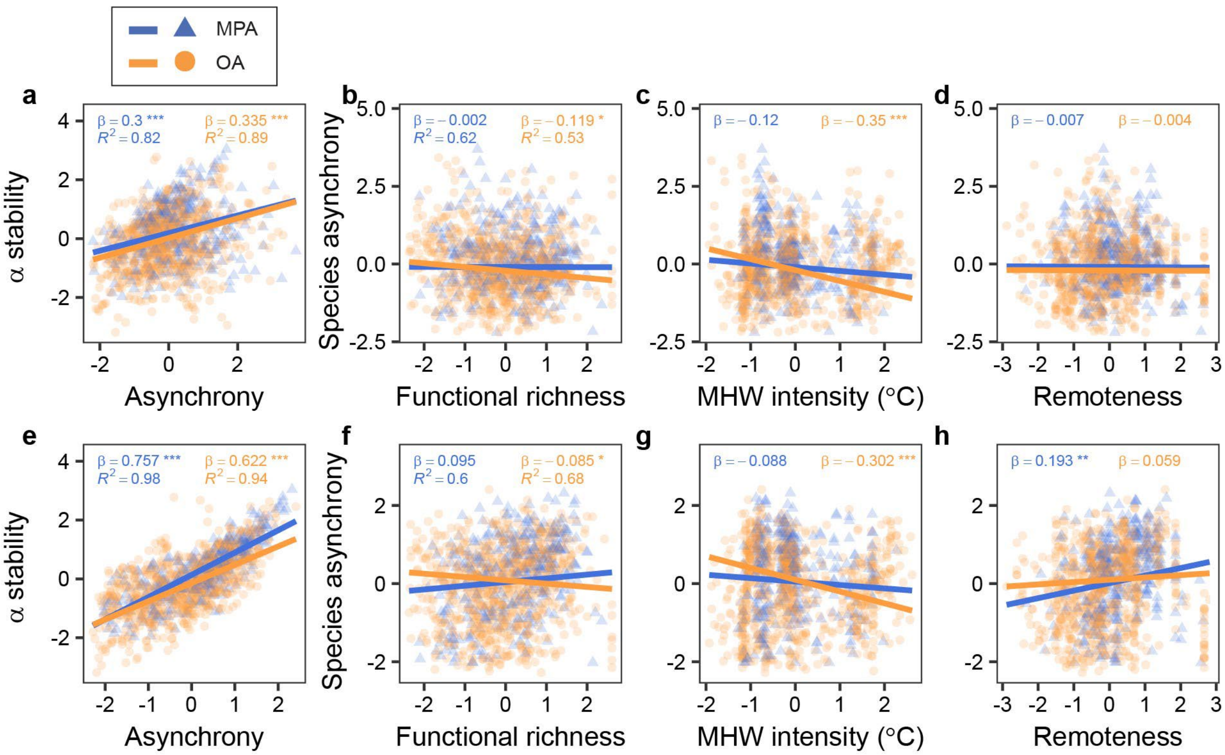

Supplementary Fig. 3. Relationships involving asynchrony either as a predictor or response variable based on (**a-d**) linearly detrended timeseries and (**e-h**) Loreau and de Mazancourt synchrony measure. Asynchrony was obtained by subtracting synchrony from unity (see Methods for details). Data are shown as z-scores for marine protected areas (MPA, blue lines and symbols) and open areas (OA, orange lines and symbols). Panels include the regression parameters estimated from the Linear Mixed Effect Models (as in Supplementary Table 1), their significance (***, $p<0.001$; **, $p<0.01$; *, $p<0.05$) and the conditional coefficients of determination, which accounts for both fixed and random effects (R^2, indicated only in the first panel for each response variable).

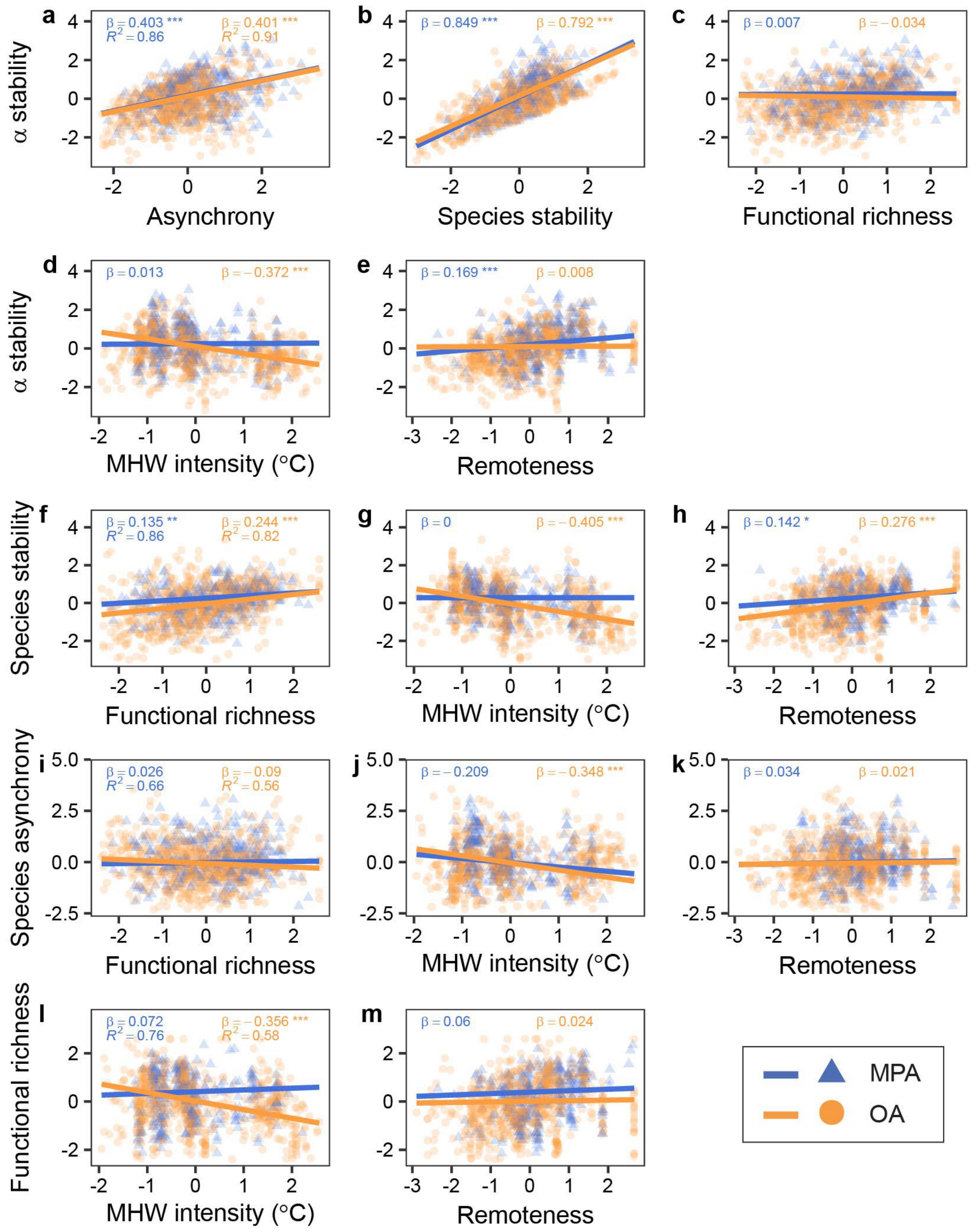

Supplementary Fig. 4. Same analysis as in Fig. 2b-k of main text and in Supplementary Fig. 1a-c, but using cumulative instead of mean intensity to characterize MHWs. Data are shown as z-scores for marine protected areas (MPA, blue lines and symbols) and open areas (OA, orange lines and symbols). Panels include the regression parameters estimated from the Linear Mixed Effect Models, their significance (***, p<0.001; **, p<0.01; *, p<0.05) and the conditional coefficients of determination, which accounts for both fixed and random effects (R^2, indicated only in the first panel for each response variable).

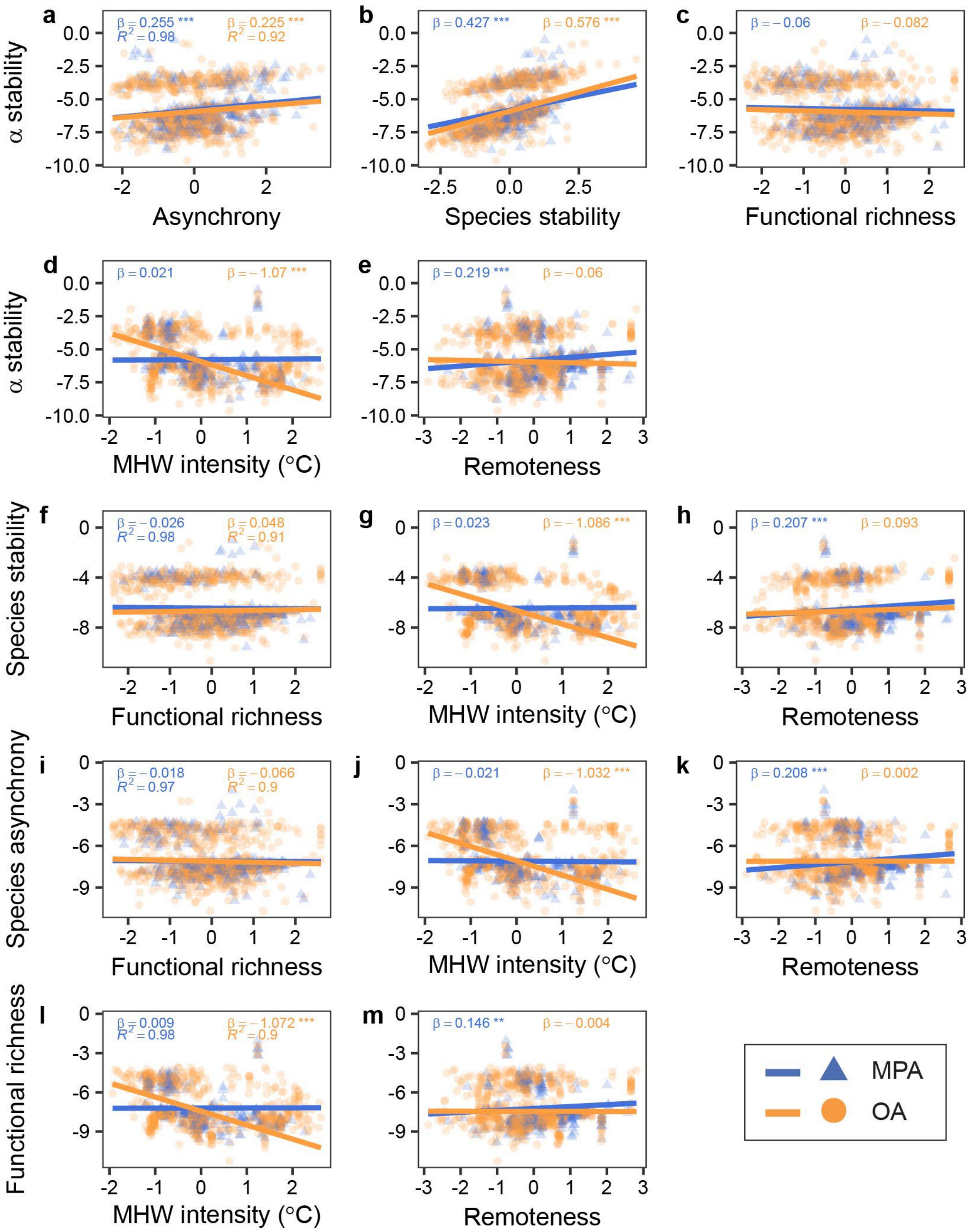

Supplementary Fig. 5. Sensitivity tests for the offset. These alternative analyses examine log-response ratios (or rates) – i.e. each response variable is divided directly by sampling effort (sampled area) and log-transformed, instead of including an offset in the Linear Mixed Effect Models, as in the main analysis. Since Gross' measure includes negative values that cannot be log-transformed, the Loreau and de Mazancourt measure of asynchrony, which varies in the range of 0-1, is used here. Results are qualitatively similar to those presented in Fig. 2b-k in the main text and in Supplementary Fig. 1, where variables are standardized after log-transformation and sampling effort is included as an offset in mixed-effect models. Data are from marine protected areas (MPA, blue lines and symbols) and open areas (OA, orange lines and symbols). Predictor variables are shown as z-scores. Panels include the regression parameters estimated from the mixed-effect models, their significance (***, p<0.001; **, p<0.01) and the conditional coefficients of determination, which accounts for both fixed and random effects (R^2, indicated only in the first panel for each response variable).

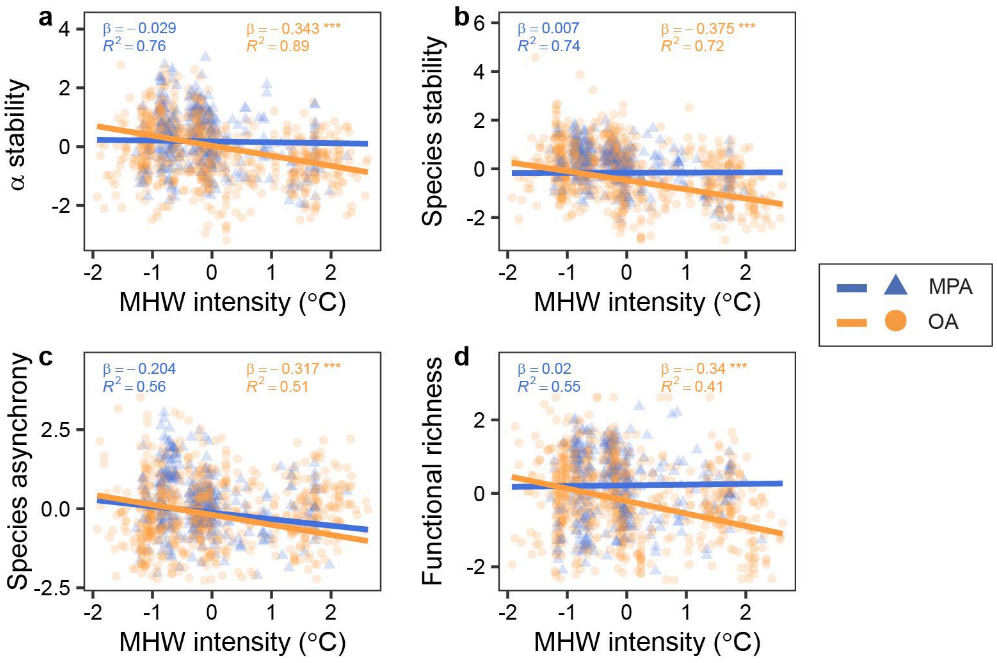

Supplementary Fig. 6. Sensitivity analysis including only sampling programs that identified more than 50 species. Relationships are shown between alpha stability (**a**), species stability (**b**), species asynchrony (**c**) and functional richness (**d**) with MHWs. Results are consistent with those reported in Fig. 2d,f,h,j of main text, which included all sampling programs. Data are shown as z-scores for marine protected areas (MPA, blue lines and symbols) and open areas (OA, orange lines and symbols). Panels include the regression parameters estimated from the Linear Mixed Effect Models, their significance (***, p<0.001) and the conditional coefficients of determination, which accounts for both fixed and random effects (R^2).

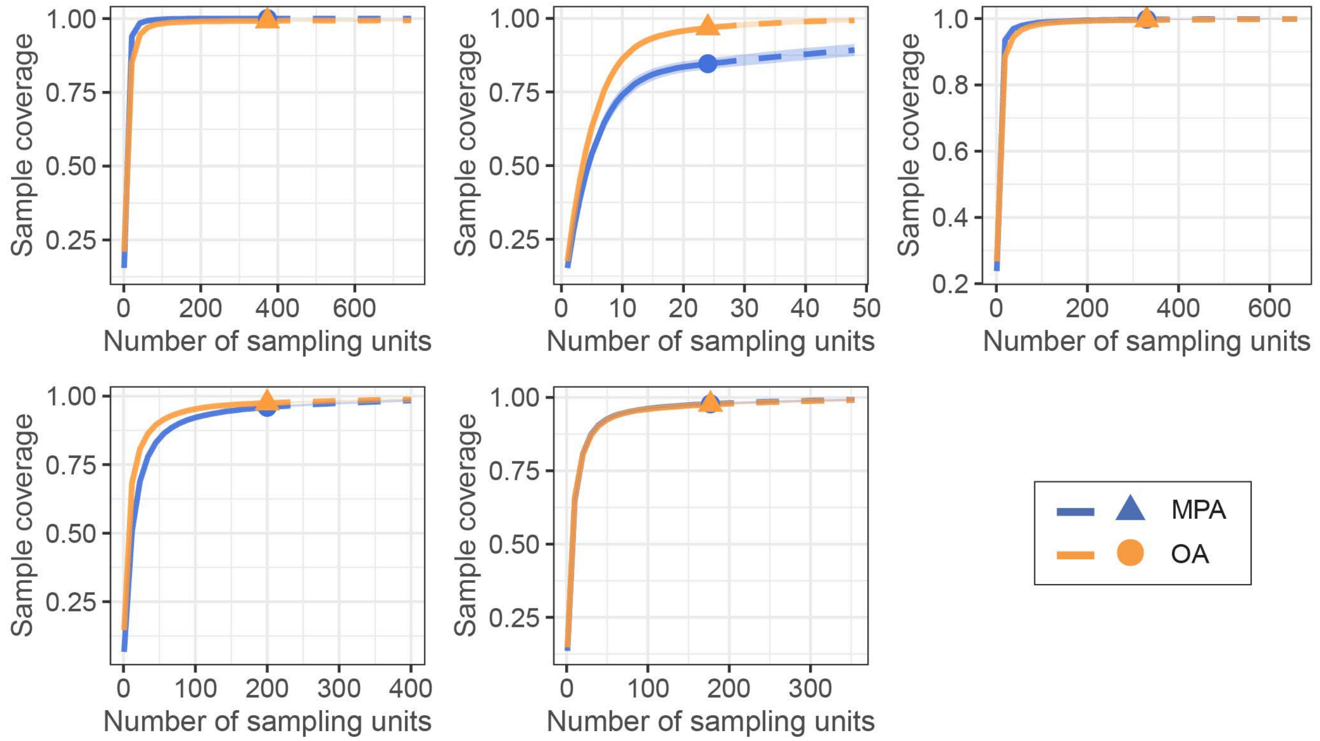

Supplementary Fig. 7. Incidence-based rarefaction-extrapolation approach to compare sample coverage between marine protected areas (MPA, blue lines and symbols) and open areas (OA, orange lines and symbols) for transects of different size (indicated in m^2 on top of the panels). Bands indicate 95% confidence intervals obtained by bootstrap (n=100).

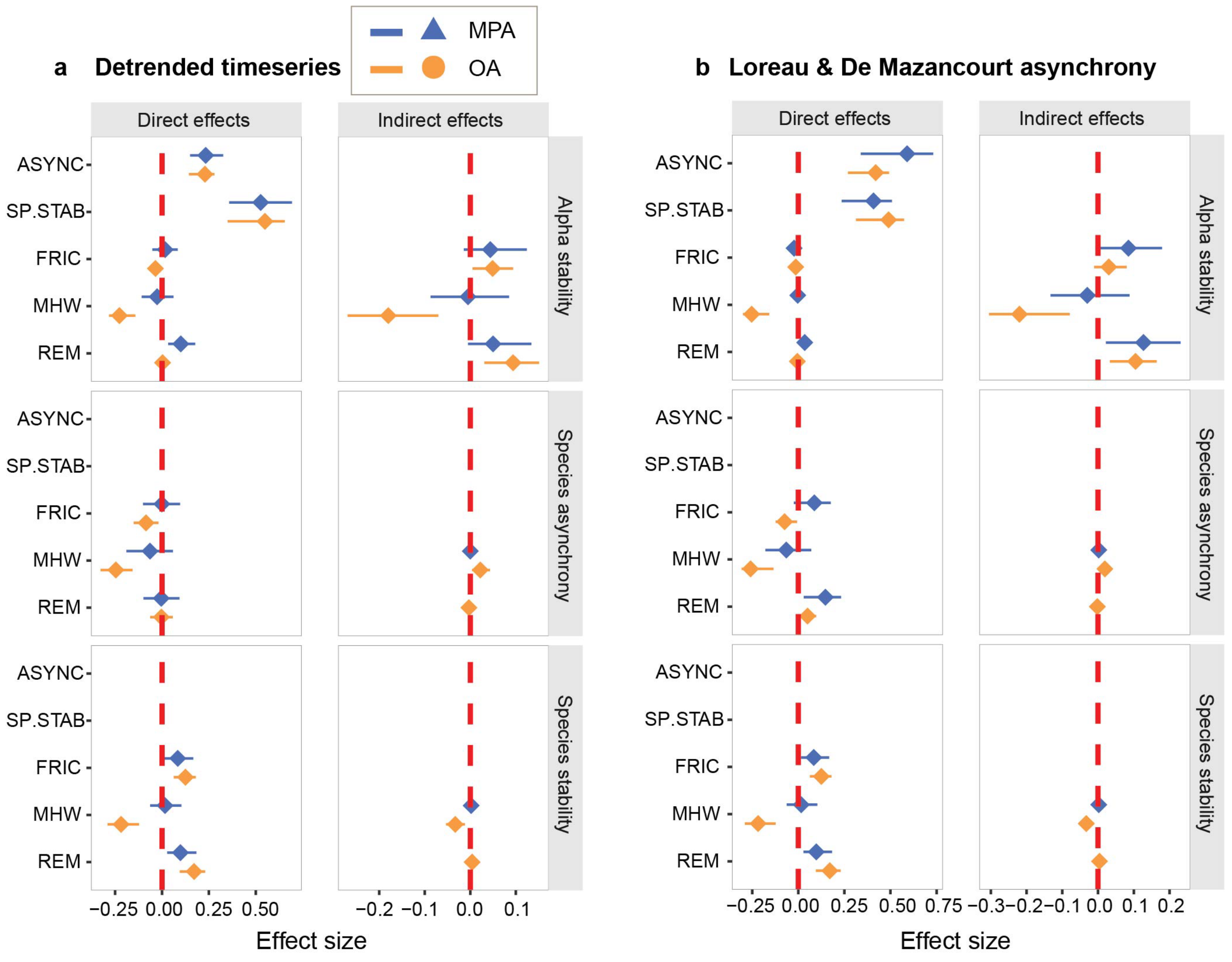

Supplementary Fig. 8. Standardized direct and indirect effect sizes (means and 95% Confidence Intervals) of factors influencing alpha and species stability and asynchrony from the piecewise Structural Equation Models reported in Fig. 3 of main text and obtained here using **a** detrended timeseries or **b** Loreau and de Mazancourt synchony measure. Data are from marine protected areas (MPA, blue lines and symbols) and open areas (OA, orange lines and symbols). ASYNC: Asynchrony; SP.STAB: species stability; FRIC: functional richness; MHW: marine heatwave intensity; REM: remoteness. Effect sizes whose confidence intervals do not overlap with zero (dashed red line) are considered significant. Positive (negative) effect sizes indicate larger (lower) stability or asynchrony in MPA than OA.

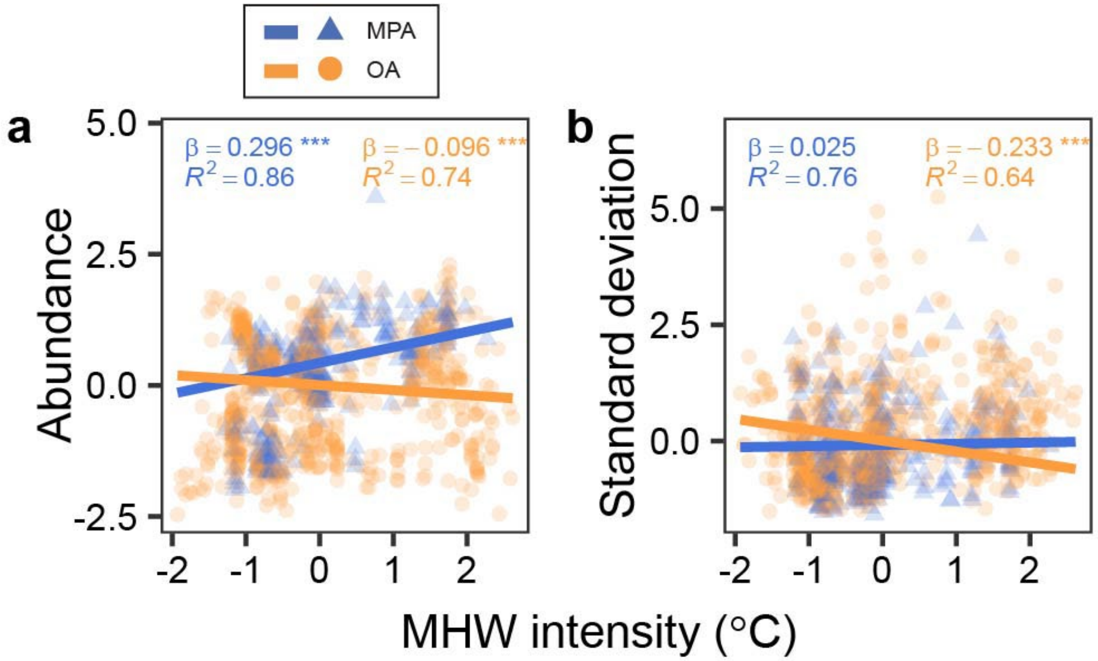

Supplementary Fig. 9. Relationships between MHWs and the two components of alpha stability: (**a**) the temporal mean (the numerator of stability) and (**b**) the temporal standard deviation (the denominator of stability) of fish abundance in each site. Trends obtained from Linear Mixed Effect Models are illustrated for marine protected areas (MPA, blue lines and symbols) and open areas (OA, orange lines and symbols). Data are shown as z-scores. Panels include the regression parameters estimated from mixed-effect models, their significance (***, $p<0.001$) and the conditional coefficients of determination, which accounts for both fixed and random effects (R^2).

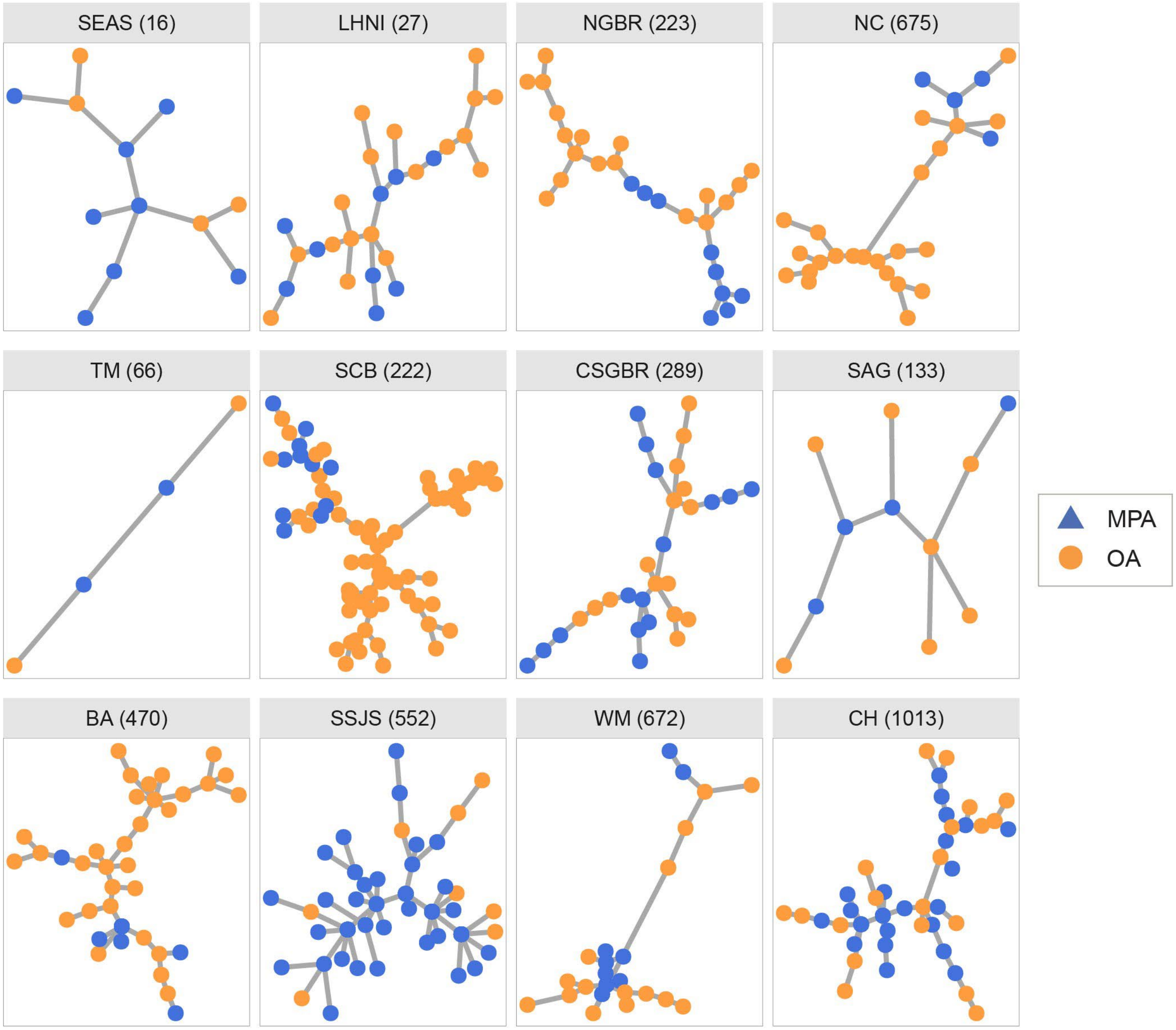

Supplementary Fig. 10. Minimum spanning tree graphs derived for each metacommunity using Jaccard dissimilarity (biological distance). The threshold distance is the minimum value of Jaccard dissimilarity that maintains all sites connected. Each panel shows sites (nodes) in marine protected areas (MPA, blue symbols) and in open areas (OA, orange symbols) linked by Jaccard dissimilarity. SEAS: South European Atlantic Shelf; LHNI: Lord Howe and Norfolk Islands; NGBR: Torres straits Norther Great Barrier Reef; NC: Norther California; TM: Tweed-Moreton; SCB: Southern California Bight; CSGBR: Central and Southern Great Barrier Reef; SAG: South Australian Gulf; BA: Bassian; SSJS: Sunda Shelf/Java Sea; WM: Western Mediterranean; CH: Cape Howe. Number within brackets next to metacommunity abbreviations indicate the spatial extent (the maximum distance among any two sites, in km) of each network.

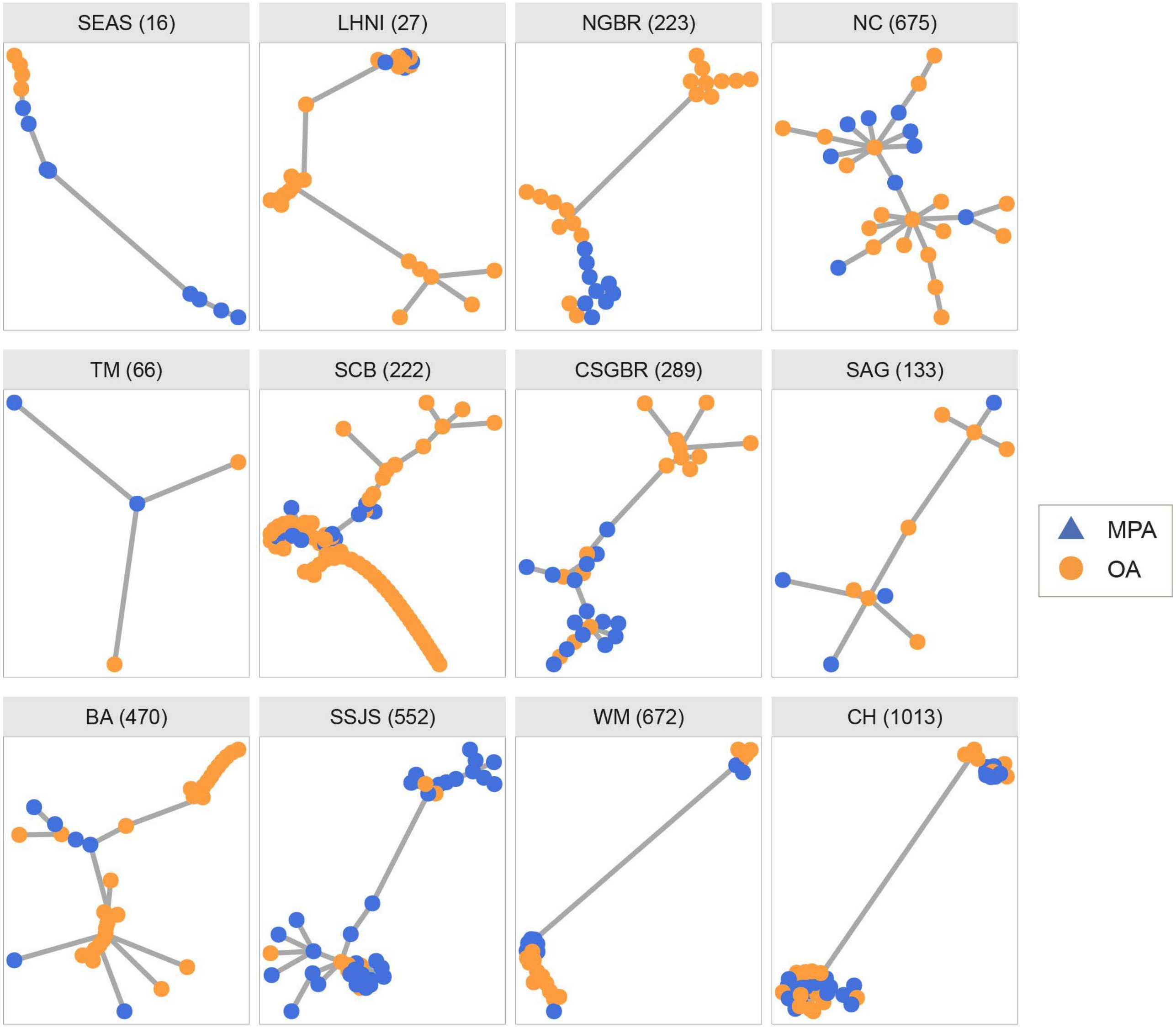

Supplementary Fig. 11. Minimum spanning tree graphs derived for each metacommunity using least-cost path distance by the sea (geographic distance). The threshold distance is the minimum physical distance that maintains all sites connected. Each panel shows sites (nodes) in marine protected areas (MPA, blue symbols) and in open areas (OA, orange symbols) linked by Jaccard dissimilarity. SEAS: South European Atlantic Shelf; LHNI: Lord Howe and Norfolk Islands; NGBR: Torres straits Norther Great Barrier Reef; NC: Norther California; TM: Tweed-Moreton; SCB: Southern California Bight; CSGBR: Central and Southern Great Barrier Reef; SAG: South Australian Gulf; BA: Bassian; SSJS: Sunda Shelf/Java Sea; WM: Western Mediterranean; CH: Cape Howe. Number within brackets next to metacommunity abbreviations indicate the spatial extent (the maximum distance among any two sites, in km) of each network.

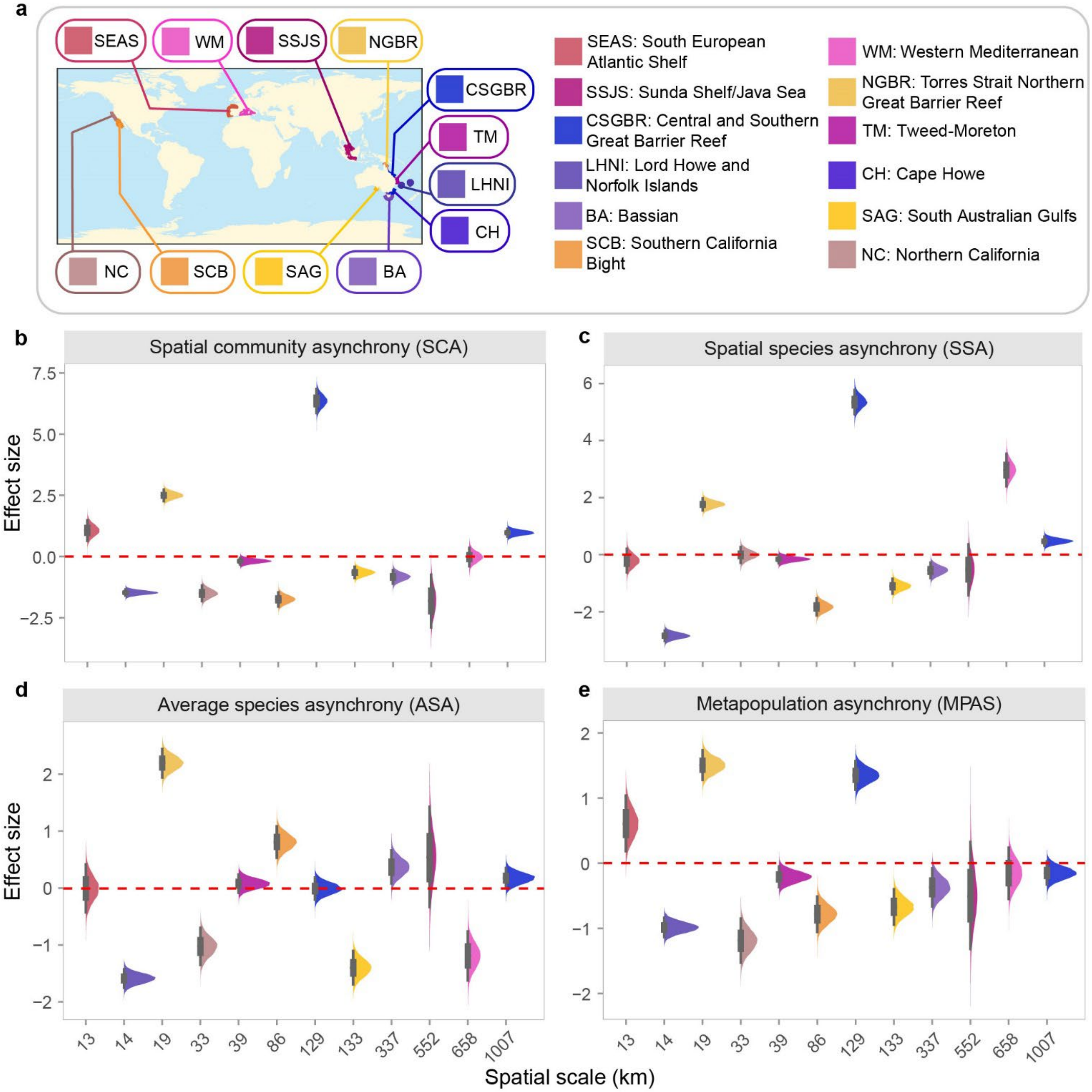

Supplementary Fig. 12. Asynchrony of marine protected area (MPA) networks. **a** Ecoregions used in the analysis of stability and asynchrony at the metacommunity level. There were at least two sites in marine protected areas (MPA) and two sites in open areas (OA) in each ecoregion (see Supplementary Table 5 for details). Panels **b-e** show the posterior distributions of effect sizes comparing different asynchrony measures between MPA and OA in relation to the spatial scale of the MPA network (see also Fig. 1 of main text). Distributions are shown with 66% (thick bar) and 95% (thin bar) uncertainty intervals. Intervals that do not overlap with 0 (dashed red line) are considered significant. Significant positive (negative) effect sizes indicate larger (lower) asynchrony in MPA than OA.

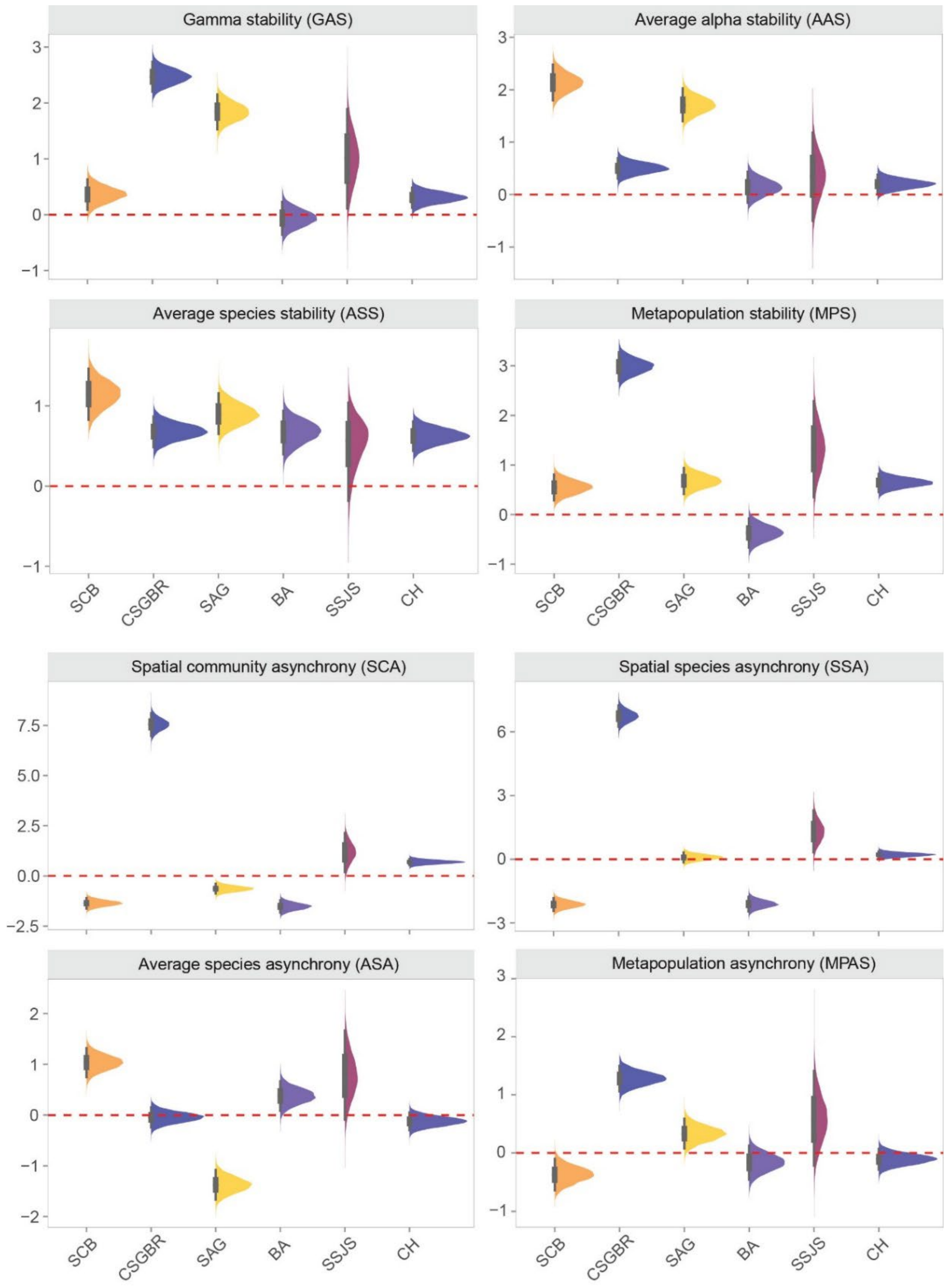

Supplementary Fig. 13. Sensitivity analysis for metacommunity stability and asynchrony measures. Panels show the posterior distributions of effect sizes comparing different stability and asynchrony measures between sites in marine protected areas (MPA) and in open areas (OA) selected within a distance range of 50-100 km. Distributions are shown with 66% (thick bar) and 95% (thin bar) uncertainty intervals. Intervals that do not overlap with 0 (dashed red line) are considered significant. Positive (negative) effect sizes indicate larger (lower) stability in MPA than OA.

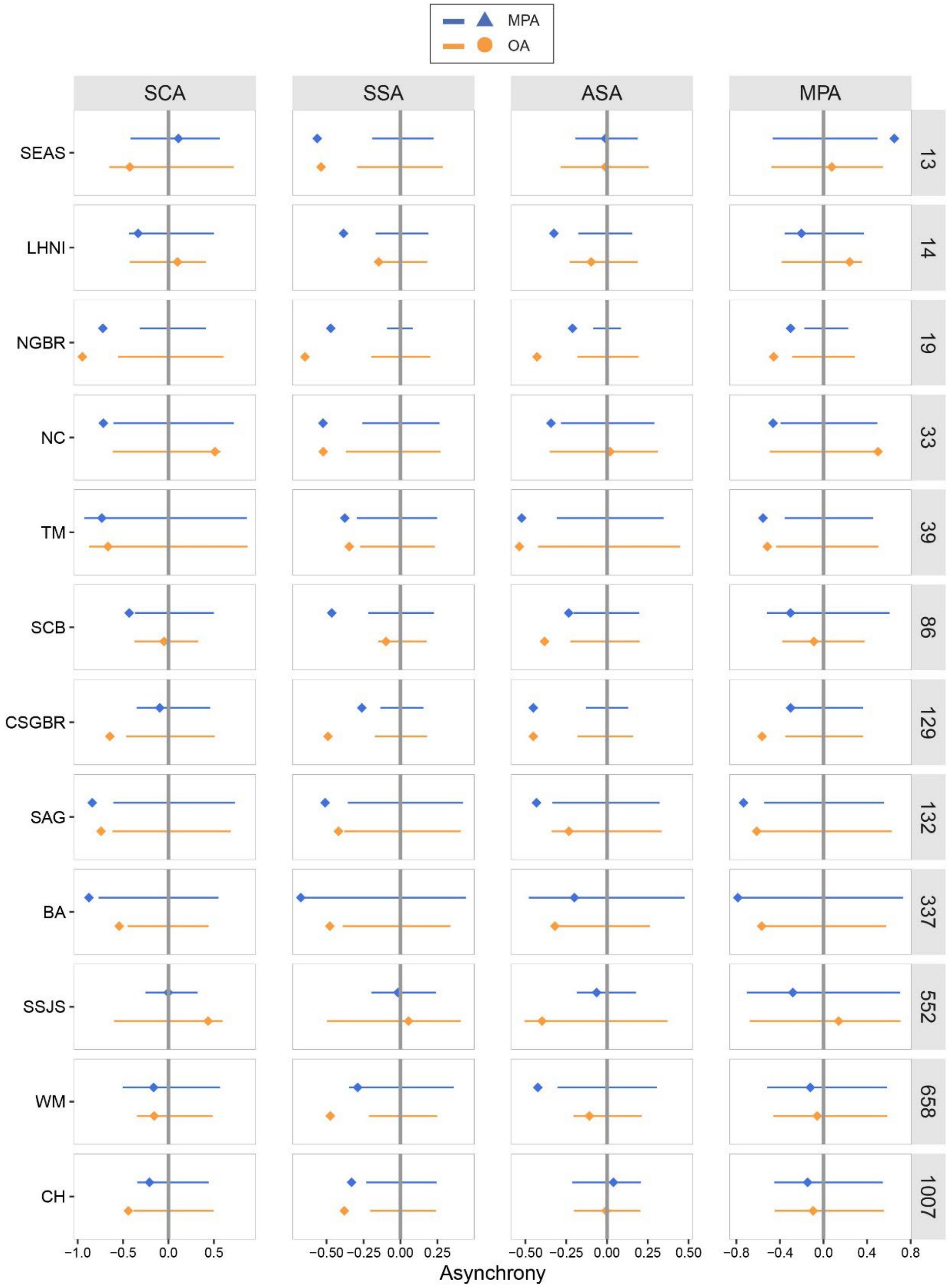

Supplementary Fig. 14. Null models of asynchrony in 12 metacommunities encompassing different spatial scales (indicated in kilometers on the right): spatial community asynchrony (SCA), spatial species asynchrony (SSA), average species asynchrony (ASA) and metapopulation asynchrony (MPAS). Symbols and lines are observed values and 95% confidence intervals (CIs), respectively, in marine protected areas (MPA, blue lines and symbols) and open areas (OA, orange lines and symbols). CIs were obtained from 999 null model simulations. Observed values outside CIs deviated significantly from null expectations. Significant positive (negative) deviations indicated larger (lower) asynchrony than expected by chance. Abbreviations of metacommunities as in Supplementary Table 5.

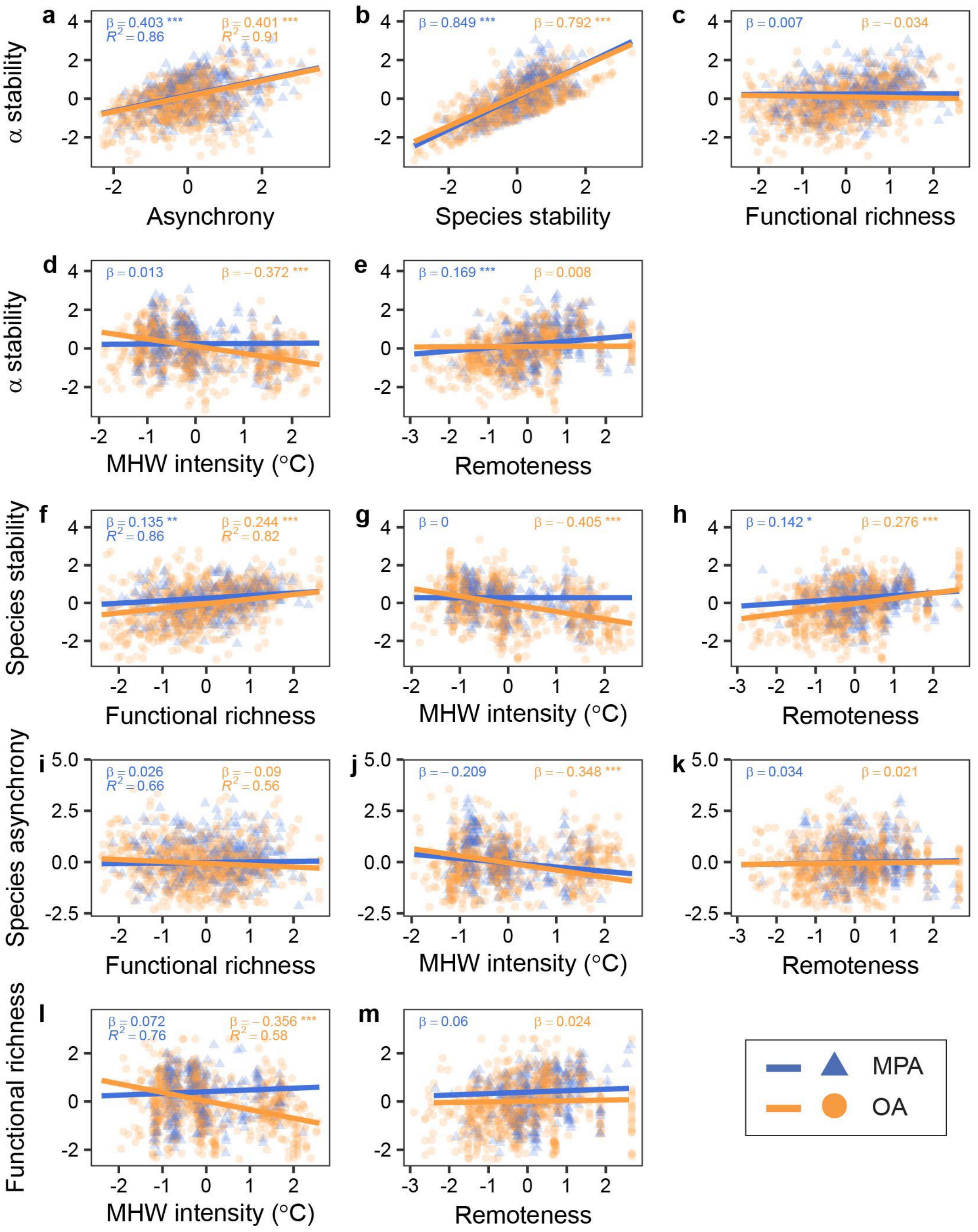

Supplementary Fig. 15. Same analysis as in Fig. 2b-k of main text and in Supplementary Fig. 1a-c, but excluding the 40 sites where all marine heatwaves were detected before the end of the climatology period (31-12-2011). Data are shown as z-scores for marine protected areas (MPA, blue lines and symbols) and open areas (OA, orange lines and symbols). Panels include the regression parameters estimated from the Linear Mixed Effect Models, their significance (***, p<0.001; **, p<0.01; *, p<0.05) and the conditional coefficients of determination, which accounts for both fixed and random effects (R^2, indicated only in the first panel for each response variable).

Supplementary Table 1. Results of Linear Mixed Effect Models on alpha stability, species stability and species asynchrony, separately for marine protected areas (MPA) and open areas (OA). These are the same models used to fit piecewise Structural Equations. ***, p<0.001; **, p<0.01; τ_{00}: random intercept for study ID; σ^2: residual variance.

	**MPA**			**OA**		
	β	*SE*	*t*	*β*	*SE*	*t*
**α STABILITY**						
Intercept	-0.13	0.31	-0.4	0.09	0.32	0.3
Asynchrony	0.41	0.03	12.4***	0.40	0.02	20.2***
Species stability	0.87	0.05	19.0***	0.81	0.03	31.4***
Functional richness	0.01	0.04	0.2	-0.05	0.02	-2.0
Marine heatwaves	-0.00	0.06	-0.1	-0.35	0.03	-13.4***
Remoteness	0.15	0.05	3.2**	0.00	0.02	0.2
**Variance**						
τ_{00} = 1.01				τ_{00} = 1.70		
σ^2 = 0.28				σ^2 = 0.28		
**SPECIES STABILITY**						
Intercept	0.05	0.47	0.1	-0.12	0.44	-0.3
Functional richness	0.12	0.05	2.5*	0.21	0.04	5.5***
Marine heatwaves	0.03	0.08	0.4	-0.37	0.04	-8.5***
Remoteness	0.17	0.06	3.0**	0.30	0.04	8.0***
**Variance**						
τ_{00} = 2.39				τ_{00} = 3.25		
σ^2 = 0.40				σ^2 = 0.80		
**SPECIES ASYNCHRONY**						
Intercept	-0.17	0.37	-0.5	-0.04	0.28	-0.2
Functional richness	0.02	0.07	0.3	-0.11	0.05	-2.3**
Marine heatwaves	-0.19	0.11	-1.73	-0.32	0.05	-6.1***
Remoteness	0.02	0.08	0.3	0.01	0.05	0.2
**Variance**						
τ_{00} = 1.37				τ_{00} = 1.17		
σ^2 = 0.81				σ^2 = 1.15		
**FUNCTIONAL RICHNESS**						
Intercept	0.27	0.37	0.7	-0.01	0.27	-0.0
Marine heatwaves	0.05	0.08	0.6	-0.34	0.05	-7.4***
Remoteness	0.03	0.06	0.5	0.04	0.04	1.1
**Variance**						
τ_{00} = 1.39				τ_{00} = 1.13		
σ^2 = 0.47				σ^2 = 0.9		

Supplementary Table 2. Results of Linear Mixed Effect Models on alpha stability. Separate models are fitted for each independent variable in interaction with protection (MPA). ***, *p*<0.001; **, *p*<0.01; *, *p*<0.05. τ_{00}: random intercept for study ID; σ^2: residual variance.

	Alpha stability			
	β	*SE*	*t*	*Variance*
**SPECIES ASYNCHRONY (AS)**				
Intercept	-0.28	0.33	-0.9	τ_{00} = 1.75
MPA	0.27	0.06	4.4***	σ^2 = 0.79
AS	0.38	0.03	11.4***	
MPA*AS	-0.05	0.06	-0.9	
**SPECIES STABILITY (SS)**				
Intercept	-0.09	0.26	-0.4	τ_{00} = 1.08
MPA	0.19	0.05	3.9***	σ^2 = 0.48
SS	0.84	0.03	29.7***	
MPA*SS	-0.03	0.06	-0.6	
**FUNCTIONAL RICHNESS (FR)**				
Intercept	-0.29	0.31	-0.9	τ_{00} = 1.53
MPA	0.34	0.07	5.2***	σ^2 = 0.90
FR	0.17	0.04	4.5***	
MPA*FR	-0.15	0.07	-2.2*	
**MHWs**				
Intercept	-0.12	0.35	- 0.3	τ_{00} = 2.05
MPA	0.31	0.06	-5.0***	σ^2 = 0.72
MHWs	-0.48	0.04	-12.2***	
MPA* MHWs	0.34	0.07	4.6***	
**REMOTENESS (RM)**				
Intercept	-0.17	0.33	-0.5	τ_{00} = 2.01
MPA	0.33	0.06	5.2***	σ^2 = 0.77
RM	0.38	0.03	10.9***	
MPA*RM	-0.27	0.07	-3.8***	

Supplementary Table 3. Results of Linear Mixed Effect Models on species stability, species asynchrony and functional richness. Separate models are fitted for each independent variable in interaction with protection (MPA). ***, p<0.001; **, p<0.01; *, p<0.05. τ_{00}: random intercept for study ID; σ^2: residual variance.								
	**Species stability**				**Asynchrony**			
	β	*SE*	*t*	*Variance*	*β*	*SE*	*t*	*Variance*
**FUNCTIONAL RICHNESS (FR)**								
Intercept	-0.44	0.37	-1.2	τ_{00} = 2.22	-0.27	0.24	-1.2	τ_{00} = 0.87
MPA	0.29	0.06	4.5**	σ^2 = 0.84	0.30	0.07	4.1***	σ^2 =1.13
FR	0.27	0.04	7.1***		-0.07	0.04	-1.7	
MPA*FR	-0.23	0.06	-3.6***		-0.12	0.07	-1.7	
**MHWs**								
Intercept	-0.27	0.41	-0.7	τ_{00} = 2.72	-0.10	0.30	-0.3	τ_{00} = 1.38
MPA	0.26	0.06	4.3***	σ^2 = 0.76	0.24	0.07	3.4***	σ^2 =1.07
MHWs	-0.51	0.04	-13.3***		-0.31	0.04	-7.0***	
MPA* MHWs	0.40	0.07	5.7***		-0.16	0.08	-1.9	
**REMOTENESS (RM)**								
Intercept	-0.17	0.33	-0.5	τ_{00} = 1.72	-0.25	0.25	-1.0	τ_{00} = 0.97
MPA	0.33	0.06	5.2***	σ^2 = 0.82	0.31	0.07	4.1***	σ^2 =1.13
RM	0.38	0.03	10.9***		0.11	0.04	2.8**	
MPA*RM	-0.27	0.07	-3.8***		-0.16	0.08	-1.9	
	**Functional richness**							
	β	*SE*	*t*	*Variance*				
**MHWs**								
Intercept	-0.02	0.27	-0.1	τ_{00} = 1.17				
MPA	0.13	0.06	2.1*	σ^2 = 0.76				
MHWs	-0.34	0.04	-8.9***					
MPA* MHWs	0.31	0.07	4.3***					
**REMOTENESS (RM)**								
Intercept	-0.10	0.27	-0.4	τ_{00} = 1.15				
MPA	0.15	0.06	2.4*	σ^2 = 0.80				
RM	0.15	0.03	4.3***					
MPA*RM	-0.19	0.07	-2.7**					

Supplementary Table 4. Results of Generalized Additive Mixed Models comparing trends of species abundance against mean intensity of marine heatwaves (MHWs) between marine protected areas (MPA) and open areas (OA). Models include a linear term to compare MPA vs. OA conditions and a tensor product for the interaction between MHWs and the linear term. Separate models are fitted for species with thermal affinities above or below threshold (maximum MHW intensity) within four fish trophic categories. SE: standard error; edf: effective degrees of freedom; significance of model term is assessed using a 2-tailed Wald test statistic. ***, *p*<0.001; **, *p*<0.01; *, *p*<0.05.

	**Above threshold**		**Below threshold**	
**CARNIVORES**				
**Linear terms**	*β (SE)*	*t*	*β (SE)*	*t*
Intercept	0.52 (0.44)	1.2	0.81 (0.53)	1.5
MPA	0.14 (0.04)	3.7***	0.31 (0.04)	7.3***
**Smooth terms**	*edf*	*Wald*	*edf*	*Wald*
MHW * MPA unprotected	4.00	15873***	3.948	20523***
MHW + MPA protected	4.00	10564***	3.853	615*
Study ID	15.90	246***	15.92	224***
R^2	0.34		0.13	
**GRAZERS**				
**Linear terms**	*β (SE)*	*t*	*β (SE)*	*t*
Intercept	-0.13 (0.30)	-0.45	-0.05 (0.25)	-0.2
MPA	0.25 (0.04)	6.5***	0.294 (0.04)	7.5***
**Smooth terms**	*edf*	*Wald*	*edf*	*Wald*
MHW * MPA unprotected	4.00	3697***	3.284	126**
MHW + MPA protected	3.98	9702***	3.934	2182***
Study ID	12.36	90***	11.22	145***
R^2	0.38		0.30	
**MICROPHAGES**				
**Linear terms**	*β (SE)*	*t*	*β (SE)*	*t*
Intercept	-1.20 (0.50)	-2.5*	-0.47 (0.74)	-0.7
MPA	0.30 (0.05)	6.11***	-0.023 (0.06)	-0.4
**Smooth terms**	*edf*	*Wald*	*edf*	*Wald*
MHW * MPA unprotected	3.952	1831***	3.906	97
MHW + MPA protected	3.958	4383***	3.837	86*
Study ID	7.65	30***	7.66	10***
R^2	0.33		0.11	
**PLANKTIVORES**				
**Linear terms**	*β (SE)*	*t*	*β (SE)*	*t*
Intercept	-0.65 (0.29)	-2.2*	-0.06 (0.52)	-0.2
MPA	0.30 (0.05)	6.4***	0.12 (0.04)	2.7**
**Smooth terms**	*edf*	*Wald*	*edf*	*Wald*
MHW * MPA unprotected	3.896	471***	3.899	177
MHW + MPA protected	3.885	1076***	3.676	1539***
Study ID	12.38	28***	13.84	98***
R^2	0.22		0.41	

Supplementary Table 5. Characteristics of marine protected area (MPA) networks used in the metacommunity analysis. MPA scale is the maximum least-cost path distance by the sea between the two MPA sites further apart. Maximum distance refers to the distance between the two sites further apart in the network, including both MPA and open areas (OA). R: rocky reef habitat; C: coral reef habitat. Average dissimilarity is the mean Jaccard index of compositional differences of fish communities between all pairs of sites in each metacommunity.

Ecoregion	MPA scale (km)	Maximum distance (km)	Number of MPA	Number of MPA sites (average dissimilarity)	Number of OA sites (average dissimilarity)	Number of years (period)
SEAS (R)	13	16	1	8 (0.31)	4 (0.34)	5 (2009-2015)
LHNI (R)	14	27	1	9 (0.61)	16 (0.57)	5 (2010-2020)
NGBR (C)	19	223	1	9 (0.45)	3 (0.38)	8 (1995-2020)
NC (R)	33	675	1	4 (0.61)	7 (0.38)	5 (2008-2017)
TM (C)	39	66	1	2 (0.61)	2 (0.56)	5 (2009-2019)
SCB (R)	86	222	1	11 (0.74)	58 (0.43)	5 (2008-2012)
CSGBR (C)	129	289	1	15 (0.40)	9 (0.45)	5 (2005-2020)
SAG (R)	133	133	1	4 (0.49)	7 (0.46)	5 (2016-2020)
BA (R)	337	470	3	6 (0.62)	31 (0.55)	5 (2006-2019)
SSJS (C)	552	552	12	35 (0.21)	8 (0.26)	5 (2014-2018)
WM (R)	658	672	3	9 (0.43)	15 (0.37)	5 (2008-2020)
CH (R)	1007	1013	3	22 (0.67)	20 (0.64)	5 (2005-2017)

SEAS: South European Atlantic Shelf; LHNI: Lord Howe and Norfolk Islands; NGBR: Torres straits Norther Great Barrier Reef; NC: Norther California; TM: Tweed-Moreton; SCB: Southern California Bight; CSGBR: Central and Southern Great Barrier Reef; SAG: South Australian Gulf; BA: Bassian; SSJS: Sunda Shelf/Java Sea; WM: Western Mediterranean; CH: Cape Howe.

Supplementary Table 6. Linear regression of degree centrality obtained from biologically-derived minimum spanning tree graphs (Jaccard dissimilarity) against closeness centrality obtained from physically-derived minimum spanning tree graphs (least-cost path distance by the sea). Significant relationships were expected for dispersal-limited communities. The lack of significant relationships suggests that physical isolation does not preclude biological connectivity.

Metacommunity	*β*	*S.E.*	*t*	*P*
SEAS	1.230	0.919	1.3	>0.2
LHNI	0.548	0.508	1.1	>0.2
NGBR	0.099	0.500	0.2	>0.8
NC	-0.188	0.541	-0.3	>0.7
TM	0.652	0.823	0.8	>0.5
SCB	-0.010	0.233	-0.4	>0.6
CSGBR	-0.083	0.476	-0.2	>0.8
SAG	1.010	0.948	1.1	>0.3
BA	0.327	0.716	0.5	>0.6
SSJS	-0.706	0.756	-0.9	>0.3
WM	0.095	0.365	0.3	>0.7
CH	0.027	0.460	0.1	>0.9

Metacommunity abbreviations as in Table 5.

Supplementary Table 7. Linear regression of *Hedge's g* effect sizes comparing metacommunity stability and asynchrony between marine protected (MPA) and open areas against three attributes of MPA networks: spatial scale, number of MPA and number of sampled sites. Only the spatial scale defined by the maximum distance between any two MPA in an ecoregion is considered here (comparisons in the distance range of 50-100 km could be made only for 6 metacommunities).

Response variable	MPA network attribute					
	Spatial scale		Number of MPAs		Number of sites	
	β (S.E.)	R^2	β (S.E.)	R^2	β (S.E.)	R^2
GAS	-0.0005 (0.001)	0.02	-0.006 (0.11)	0.00	-0.004 (0.02)	0.00
AAS	-0.001 (0.001)	0.19	-0.07 (0.09)	0.06	0.007 (0.02)	0.02
ASS	0.0004 (0.001)	0.01	-0.003 (0.14)	0.00	0.032 (0.05)	0.12
MPS	0.0000 (0.001)	0.00	0.03 (0.08)	0.01	0.006 (0.02)	0.01
SCA	-0.0003 (0.0028)	0.00	-0.22 (0.23)	0.08	-0.04 (0.05)	0.07
SSA	0.0013 (0.0021)	0.04	-0.04 (0.22)	0.004	-0.03 (0.04)	0.04
ASA	-0.0000 (0.0011)	0.00	0.06 (0.11)	0.03	0.016 (0.02)	0.06
MPA	-0.0002 (0.001)	0.01	-0.04 (0.09)	0.02	-0.015 (0.02)	0.07

GAS: gamma stability; AAS: average alpha stability; ASS: average species stability; MPS: metapopulation stability; SCA: spatial community asynchrony; SSA: spatial species asynchrony; ASA: average species asynchrony; MPA: metapopulation asynchrony.

Supplementary Table 8. Data sources, links and spatiotemporal characteristics. Each data source includes multiple sites that are not necessarily sampled in the same years. Column "Years" indicates the first and the last year included in each data source. The longest timeseries available (the site with the maximum number of sampling years) is indicated for each data source in column "Longest timeseries". MPA: marine protected areas; OA: open areas.

Data source	Link	N. MPA	N. MPA Sites	N. OA Sites	Years	Longest time-series
Online databases						
Reef Life Survey	https://reeflifesurvey.com/	23	52	130	2006-2020	13
Reef Check	https://www.reefcheck.org/	74	70	264	1997-2019	19
Published dataset						
BioTIME	https://onlinelibrary.wiley.com/doi/10.1111/geb.12729	[§]0	0	96	1997-2020	21
Western Central Pacific	https://www.nature.com/articles/sdata201717 6	4	6	6	2010-2017	6
Monitoring programs						
Santa Barbara Channel	Kushner, J.D. DavidKushnerKFMP@gmail.com Reed, D. danreed@ucsb.edu	1	15	18	2003-2019	17
Great Barrier Reef Marine Park	Emslie, M. M.Emslie@aims.gov.au	1	123	140	1994-2021	19
Portugal	Costa e Horta, B.bbcosta@ualg.pt Gonçalves, J. E. emanuel@ispa.pt	1	8	4	2009-2015	5
Southern Australia	Barrett, N. neville.barrett@utas.edu.au Edgar, G. g.edgar@utas.edu.au	10	73	71	1992-2020	28
Western Mediterranean	García Charton, J.A. jcharton@um.es Aspillaga, E. aspillaga@imedea.uib-csic.es Hereu, B. hereu@ub.edu	5	10	18	1996-2020	19

[§]Although the BioTIME database included only open areas, these data were part of the sampling programs maintained in 6 marine protected areas and were therefore included in alpha and gamma stability analyses.
