# Supplementary material for: Marine protected areas promote stability of reef fish communities under climate warming
Source: Nat Commun. 2024 Feb 28;15:1822. doi: 10.1038/s41467-024-44976-y (PMC10902350; doi:10.1038/s41467-024-44976-y)
Supplement: Supplementary file 3 — Reporting Summary [file 41467_2024_44976_MOESM3_ESM.pdf]

nature portfolio

Corresponding author(s): Lisandro Benedetti-Cecchi

Last updated by author(s): Dec 23, 2023

Reporting Summary

Nature Portfolio wishes to improve the reproducibility of the work that we publish. This form provides structure for consistency and transparency in reporting. For further information on Nature Portfolio policies, see our Editorial Policies and the Editorial Policy Checklist.

Statistics

For all statistical analyses, confirm that the following items are present in the figure legend, table legend, main text, or Methods section.

n/a	Confirmed	
☐	☒	The exact sample size (n) for each experimental group/condition, given as a discrete number and unit of measurement
☐	☒	A statement on whether measurements were taken from distinct samples or whether the same sample was measured repeatedly
☐	☒	The statistical test(s) used AND whether they are one- or two-sided<br>*Only common tests should be described solely by name; describe more complex techniques in the Methods section.*
☐	☒	A description of all covariates tested
☐	☒	A description of any assumptions or corrections, such as tests of normality and adjustment for multiple comparisons
☐	☒	A full description of the statistical parameters including central tendency (e.g. means) or other basic estimates (e.g. regression coefficient) AND variation (e.g. standard deviation) or associated estimates of uncertainty (e.g. confidence intervals)
☐	☒	For null hypothesis testing, the test statistic (e.g. F, t, r) with confidence intervals, effect sizes, degrees of freedom and P value noted<br>*Give P values as exact values whenever suitable.*
☐	☒	For Bayesian analysis, information on the choice of priors and Markov chain Monte Carlo settings
☒	☐	For hierarchical and complex designs, identification of the appropriate level for tests and full reporting of outcomes
☐	☒	Estimates of effect sizes (e.g. Cohen's d, Pearson's r), indicating how they were calculated

Our web collection on statistics for biologists contains articles on many of the points above.

Software and code

Policy information about availability of computer code

Data collection	Data were initially stored in Excel files by data curators and then imported in the Open Source R Computational Environment (4.1.3) as .RData files
Data analysis	All analyses were performed in R 4.1.3 using the following packages: brms 2.16.3, broom 0.8.0, codyn 2.0.5, datawizard 0.6.1, DHARMa 0.4.5, doMC 1.3.5, FD 1.0.12.1, fishualize 0.2.2, foreach 1.5.2, ggeffects 1.1.1, ggstatsplot 0.9.4, igraph 1.2.9, knitr 1.39, lemon 0.4.5, lme4 1.1.30, lmerTest 3.1.3, mFD 1.0.1, mgcv 1.8.38, modelr 0.1.8.9000, performance 0.9.2.4, piecewiseSEM 2.2.0, rnaturalearth 0.1.0, semEff 0.6.1, sf 1.0.8, sjPlot 2.8.10, tidybayes 3.0.2.9000, tidyverse 1.3.1, tidymv 3.3.1.

For manuscripts utilizing custom algorithms or software that are central to the research but not yet described in published literature, software must be made available to editors and reviewers. We strongly encourage code deposition in a community repository (e.g. GitHub). See the Nature Portfolio guidelines for submitting code & software for further information.

Data

Policy information about availability of data

All manuscripts must include a data availability statement. This statement should provide the following information, where applicable:

- Accession codes, unique identifiers, or web links for publicly available datasets
- A description of any restrictions on data availability
- For clinical datasets or third party data, please ensure that the statement adheres to our policy

All the data required to reproduce the results of this study have been deposited in the Figshare database under accession code https://figshare.com/s/ffa4f5cb22799532bbc1 and on Github at https://github.com/bencecc/ReefFishStability105. Global SST data can be accessed at https://www.ncei.noaa.gov/data/sea-surface-temperature-optimum-interpolation/v2.1/access/avhrr/. Fish abundance data were obtained from: Reef Life Survey (https://reeflifesurvey.com/), Reef Check (https://www.reefcheck.org/), BioTime (https://onlinelibrary.wiley.com/doi/10.1111/geb.12729), the Long-Term Monitoring of Coral Reef Fish Assemblages in the Western Pacific (https://www.nature.com/articles/sdata2017176). Additional data were provided by co-authors DJK, DCR, MJE, BHC, EJG, NSB, GJE, JAGC, EA, BH.

Research involving human participants, their data, or biological material

Policy information about studies with human participants or human data. See also policy information about sex, gender (identity/presentation), and sexual orientation and race, ethnicity and racism.

Reporting on sex and gender	NA
Reporting on race, ethnicity, or other socially relevant groupings	NA
Population characteristics	NA
Recruitment	NA
Ethics oversight	NA

Note that full information on the approval of the study protocol must also be provided in the manuscript.

Field-specific reporting

Please select the one below that is the best fit for your research. If you are not sure, read the appropriate sections before making your selection.

☐ Life sciences ☐ Behavioural & social sciences ☒ Ecological, evolutionary & environmental sciences

For a reference copy of the document with all sections, see nature.com/documents/nr-reporting-summary-flat.pdf

Ecological, evolutionary & environmental sciences study design

All studies must disclose on these points even when the disclosure is negative.

Study description	Data consisted of timeseries of fish abundance collected at multiple sites inside (Marine Protected Areas, MPAs) and outside (Opena Areas, OAs) marine protected areas. Protection was coded as a fixed factor with two levels (MPA vs. OA) that was crossed with several continuous covariates (e.g., Marine Heatwaves, Remoteness) to test the hypothesis that relationships of stability and asynchrony metrics derived from timeseries with covariates differed between levels of protection.
Research sample	Dataset of 71,269 timeseries of population abundances from 2,269 reef fish species sampled in 357 MPA and 747 OA sites across 50 Marine Ecoregions. Timeseries ranged from 5 to 28 years between 1992 and 2021 (February). Global SST data can be accessed at https://www.ncei.noaa.gov/data/sea-surface-temperature-optimum-interpolation/v2.1/access/avhrr/. Fish abundance data were obtained from: Reef Life Survey (https://reeflifesurvey.com/), Reef Check (https://www.reefcheck.org/), BioTime (https://onlinelibrary.wiley.com/doi/10.1111/geb.12729), the Long-Term Monitoring of Coral Reef Fish Assemblages in the Western Pacific (https://www.nature.com/articles/sdata2017176). Additional data were provided by co-authors DJK, DCR, MJE, BHC, EJG, NSB, GJE, JAGC, EA, BH.
Sampling strategy	Fish abundance was sampled by divers with visual methods. These methods fall in two categories: fish transects and cylindrical plots. Although sample size was not predetermined and sampled area differed among the sampling programs from which the dataset was assembled, we performed sample coverage analysis to show that fish communities were adequately sampled. In particular, only minimal differences in sampling accuracy were observed between MPAs and OAs for transects in the size category of 180 m^2. Removing these transects (2% of the total) did not affect the results. Differences in sampling effort were accounted for by including sampled area as an offset in statistical models.
Data collection	All data consisted of quantitative surveys of reef fish abundances obtained by a combination of marine scientists and trained

Data collection	recreational SCUBA divers, using standardized visual methods. Methodological details, data curation and diver training are provided in refs 70 and 71 and in Supplementary Table 8.
Timing and spatial scale	The analysis uses annual timeseries of fish abundance, with a minimum length of 5 years. The oldest timeseries starts in 1992 and the most recent one ends in 2021.
Data exclusions	No data were excluded from the primary analysis. For sensitivity tests, the analysis of fish stability was repeated by excluding fish abundance data from transects of 180 m^2; a second sensitivity analysis was done by excluding sites with less than 50 species.
Reproducibility	It is impossible to reproduce past data. We ensure reproducibility of the results by providing the necessary data and code to reproduce the results.
Randomization	The main cateogorization for our data is the distinction between MPA and OA sites. MPAs are not random, whereas OA sites can be considered a random sample.
Blinding	Blinding was not relevant in the study since there was no random allocation of sampling units to treatments and controls.

Did the study involve field work? ☒ Yes ☐ No

Field work, collection and transport

Field conditions	This is not possible for the 71269 timeseries used in the anlaysis. However, data on marine heatwaves, a main covariate in the study, is provided at the sites scale.
Location	Global. Geographic coordinates of sampling sites are provided in the datasets associated with this analysis.
Access & import/export	The data used in this analysis originate from two globally distributed databases, Reef Life Survey (RLS, https://reeflifesurvey.com/) and Reef Check (RC, https://www.reefcheck.org/), published datasets (refs 68,69) and scientific monitoring programs (Supplementary Table 8). All studies and programmes have their own statements of compliance with national and international regulations.
Disturbance	Fish surveys were based on non-destructive methods, resulting in no damage to fish or other organisms.

Reporting for specific materials, systems and methods

We require information from authors about some types of materials, experimental systems and methods used in many studies. Here, indicate whether each material, system or method listed is relevant to your study. If you are not sure if a list item applies to your research, read the appropriate section before selecting a response.

Materials & experimental systems

n/a	Involved in the study	
☒	☐	Antibodies
☒	☐	Eukaryotic cell lines
☒	☐	Palaeontology and archaeology
☒	☐	Animals and other organisms
☒	☐	Clinical data
☒	☐	Dual use research of concern
☒	☐	Plants

Methods

n/a	Involved in the study	
☒	☐	ChIP-seq
☒	☐	Flow cytometry
☒	☐	MRI-based neuroimaging
